# Cefepime-Induced Neurotoxicity or Nonconvulsive Status Epilepticus (NCSE): A Controversy Revisited

**DOI:** 10.7759/cureus.38050

**Published:** 2023-04-24

**Authors:** Zacharia Shebani, Brian Walter, Todd Masel, Chilvana Patel, Xiangping Li

**Affiliations:** 1 Neurology, University of Texas Medical Branch at Galveston, Galveston, USA; 2 Neurology, Houston Methodist Hospital, Houston, USA

**Keywords:** ictal-interictal continuum (iic), cefepime, nonconvulsive status epilepticus (ncse), eeg, encephalopathy

## Abstract

Neurotoxicity is a well-known side effect of cefepime among patients commonly present with altered mental status and typical electroencephalogram (EEG) findings of generalized periodic discharges (GPDs). Some practitioners consider this pattern as encephalopathy and often treat it with the withdrawal of cefepime only, while others are at times concerned with non-convulsive status epilepticus (NCSE) and treat it with antiseizure medications (ASMs) in addition to the withdrawal of cefepime to accelerate the recovery. We present a case series of two patients who developed cefepime-induced altered mental status and EEG findings of GPDs at a rate of 2-2.5 Hz concerning for the ictal-interictal continuum (IIC). Both cases were treated as possible NCSE with ASMs in addition to the withdrawal of cefepime, resulting in different clinical outcomes. The first case showed clinical and EEG improvement shortly after the administration of parenteral benzodiazepines and ASMs. The other case showed electrographic improvement but did not show significant improvement in mentation, and the patient died eventually.

## Introduction

Cefepime is a broad-spectrum, fourth-generation cephalosporin antibiotic that has been used in clinical practice because of its broad-spectrum coverage against both Gram-positive and Gram-negative bacteria. It is generally well tolerated but can be associated with neurotoxicity. Patients with underlying renal insufficiency and high cefepime plasma concentrations are at increased risk of neurotoxicity [1]. One frequently described manifestation of cefepime-induced neurotoxicity (CIN) is altered mental status with concurrent generalized periodic discharges (GPDs) on the EEG. To this date, the interpretations of this electroclinical manifestation continue to be vague and controversial [2]. Those who use the interpretation of encephalopathy often treat solely by the withdrawal of cefepime, while others who use the interpretation of NCSE are more likely to treat with the addition of antiseizure medications (ASMs) [2]. There is no consensus regarding when to treat or how aggressive the treatment should be. In our case series, we present two cases of suspected cefepime-induced NCSE treated with ASMs in addition to the withdrawal of cefepime, which resulted in different electroclinical responses. In this effort, we would like to determine the clinical scenarios where EEGs are necessary, identify some of the characteristics of the EEG findings, and demonstrate whether any treatment with ASMs could be beneficial. 

## Case presentation

Case 1

A 76-year-old woman with a past medical history of diabetes, hypertension, and dyslipidemia presented to the hospital for stercoral colitis and fecal impaction. The patient was started on empiric antibiotics, including cefepime 2000 mg Q12h, vancomycin, and metronidazole, due to fever and suspected stercoral colitis. Lab testing on admission showed mildly impaired kidney function (Table 1).

**Table 1:** Lab results. BUN, blood urea nitrogen

Lab test	Value (mg/dL)	Reference range (mg/dL)
BUN	18	7-23
Creatinine	1.15	0.50-1.04

**Figure 1:**
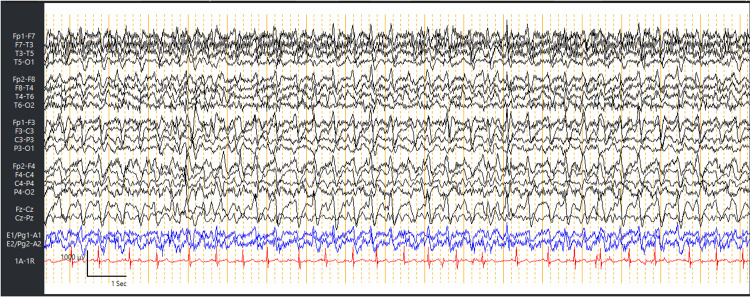
EEG demonstrated continuous GPDs with triphasic morphology at a rate of 2-2.5 Hz. EEG, electroencephalogram; GPDs, generalized periodic discharges

**Figure 2:**
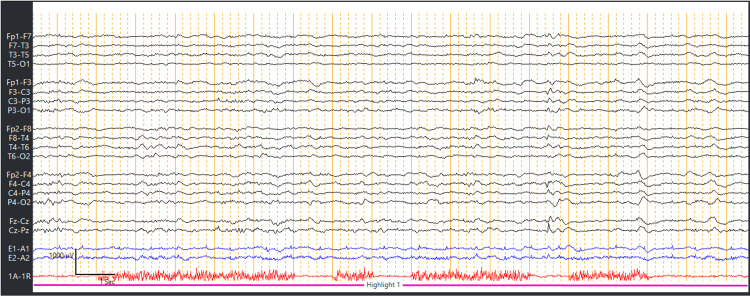
EEG showed electroencephalographic improvement, after treatment with antiepileptic medications (lorazepam and levetiracetam). EEG, electroencephalogram

On day 2 of cefepime treatment, the patient began to display intermittent myoclonus in all extremities and worsening of mentation. Neurology was consulted. The electroencephalogram (EEG) revealed continuous GPDs with triphasic morphology at a rate of 2-2.5 Hz (Figure 1). Cefepime was discontinued, and the patient was given a diagnostic trial of lorazepam 4 mg and levetiracetam 40 mg/kg. It showed significant improvement following the administration of lorazepam and levetiracetam (Figure 2). The patient’s neurological status improved on the same day, and she responded to questions appropriately. MRI brain and cerebrospinal fluid (CSF) studies were unremarkable. The patient was later discharged with a complete resolution of neurological symptoms. 

Case 2

**Figure 3:**
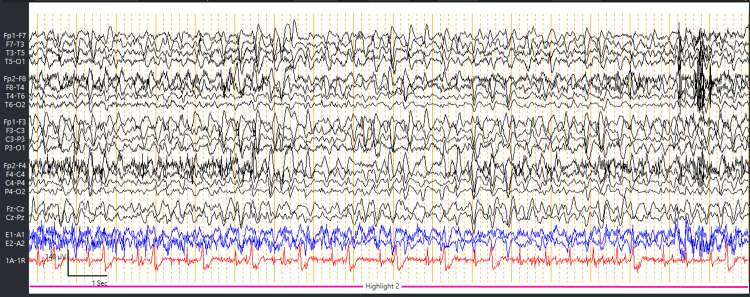
EEG showed 2-2.5 Hz generalized periodic discharges with triphasic morphology. EEG, electroencephalogram

A 95-year-old woman with a past medical history notable for chronic obstructive pulmonary disease (COPD) on oxygen supplementation, heart failure, hypertension, breast cancer s/p mastectomy, and dementia, presented to the hospital with shortness of breath and a non-productive cough. Chest X-ray findings were consistent with right lower lobe pneumonia. The patient was started on cefepime 2000 mg every 8 h, and vancomycin 750 mg every 12 h. On day three of cefepime therapy, neurology was consulted to evaluate the patient for altered mental status and global aphasia. The CT head was unremarkable. Continuous EEG initially showed near continuous 2-2.5 Hz bifrontal GPDs with triphasic morphology (Figure 3). Her renal function was normal (Table 2).

**Table 2:** Lab results. BUN, blood urea nitrogen

Lab test	Value (mg/dL)	Reference range (mg/dL)
BUN	10	7-23
Creatinine	0.47	0.50-1.04

**Figure 4:**
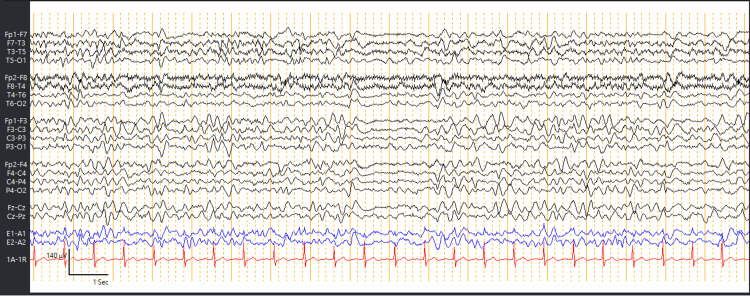
EEG after 2 h of treatment with Levetiracetam demonstrated the improvement of the generalized periodic discharges compared with the initial EEG. Benzodiazepine was given 3 days after Levetiracetam. EEG, electroencephalogram

Cefepime was discontinued, and the patient was treated with an alternative antibiotic (azithromycin and piperacillin-tazobactam). The patient was treated with a loading dose of levetiracetam of 60 mg/kg, with some improvement seen in the EEG (Figure 4). Lorazepam was given 3 days after the administration of levetiracetam. Besides a maintenance dose of levetiracetam during the hospital course, the patient was treated with lacosamide 200 mg BID for 2 weeks and phenytoin 100 mg TID for 1 week due to the presence of intermittent 1-1.5 Hz GPDs with triphasic morphology. The patient showed minimal neurological improvement despite treatment with three ASMs. After a family meeting, she was placed in hospice care and later passed away within 3 weeks of admission.

## Discussion

Cefepime is frequently reported to cause neurotoxicity. The incidence of CIN and NCSE is quite variable in the literature. A meta-analysis performed by Appa et al. gathered data from 71 individual studies with 198 subjects between January 2013 and June 2016 showed that the most common clinical manifestations of CIN reported are diminished level of consciousness (80%), agitation (47%), myoclonus (40%), non-convulsive status epilepticus (31%), seizures (11%), and aphasia (9%) [3].

The typical period for the onset of cefepime-induced encephalopathy is 1-10 days after the start of cefepime, with resolution in 2-7 days following cessation [4]. The mechanism of cefepime-induced NCSE appears to be gamma aminobutyric acid (GABA)-A receptor antagonism, reducing the GABA-mediated inhibitory response, and therefore generating pro-epileptogenic activity [5]. The term cefepime-induced NCSE is familiar to physicians, yet its clinical presentation, EEG findings, and treatment are poorly characterized and remain controversial. There is insufficient data to understand whether cefepime-induced NCSE and encephalopathy are two distinct clinical entities or whether they represent part of a continuum [3]. It is also controversial to define the EEG pattern of cefepime-induced GPDs as epileptiform or non-epileptiform.

In our study, both patients presented with acute mental status changes, neurological symptoms, and EEG findings of 2-2.5 Hz GPDS with triphasic morphology. According to the ACNS guideline [6], these periodic EEG patterns fall along the ictal-interictal continuum (IIC) and warrant a trial with the administration of a benzodiazepine or other rapidly acting ASMs [7].

In the first case, the patient developed myoclonus and an altered mental status 24 h after the initiation of treatment with cefepime. Shortly after the administration of parenteral lorazepam and levetiracetam, there was a clear clinical and electrographic improvement, thus fulfilling the criteria for definitive NCSE as per ACNS guidelines [6]. The patient had complete neurological recovery in 24 h, which supports the role of the ASM trial in management.

In the second case, the electrographic improvement without clinical improvement after treatment with ASM (levetiracetam) fulfilled the ACNS criteria for possible NCSE [6]. Lorazepam was given 3 days after the administration of levetiracetam. However, the subsequent continuous EEG showed intermittent runs of 1-2 Hz GPDs, and the additional treatment with lacosamide and phenytoin did not change the EEG or clinical status. In this case, possibly, the GPDs represent an interictal rather than an ictal pattern. Moreover, the poor outcome, and persistence of GPDs in the patient could be multifactorial and related to comorbid cardiopulmonary, neurodegenerative, and metabolic conditions, among other factors such as senility and polypharmacy. It is uncertain how aggressively to pursue treatment of these GPD patterns, particularly when aggressive treatment can be associated with adverse effects.

Neurotoxicity is directly proportional to cefepime plasma concentration. In one study, it was found that there was no risk of developing neurotoxicity with cefepime plasma trough concentrations <7.7 mg/L, but all individuals with concentrations >38.1 mg/L presented with neurological symptoms [8]. Higher cefepime levels could be treated with dialysis to accelerate improvement [8]. However, this lab order is not widely available.

Further research is needed in the field to properly and universally define EEG patterns associated with cefepime-related NCSE. There is also a need to design proper evidence-based, effective management protocols to improve clinical outcomes.

## Conclusions

Cefepime-induced NCSE is often reported in the literature, although the clinical manifestation, EEG pattern, and management protocol are not well defined. Physicians need to be aware of the risk of NCSE and carefully evaluate suspected cases through EEG monitoring and clinical judgment to help differentiate encephalopathy from NCSE. In cases with GPDs suggestive of the IIC (1-2.5 Hz), cefepime should be discontinued, and a treatment trial (typically with a parenteral benzodiazepine) could be useful. The use of maintenance ASM remains controversial, and further clinical studies are needed. 
